# "Before we used to get sick all the time": perceptions of malaria and use of long-lasting insecticide-treated bed nets (LLINs) in a rural Kenyan community

**DOI:** 10.1186/1475-2875-9-345

**Published:** 2010-11-30

**Authors:** Timothy DV Dye, Rose Apondi, Eric S Lugada, James G Kahn, Jacqueline Smith, Caroline Othoro

**Affiliations:** 1 Department of Public Health and Preventive Medicine, State University of New York Upstate Medical University. SUNY Upstate Medical University, Institute for Human Performance, 505 Irving Avenue, Room 4004, Syracuse, New York 13210, USA; 2 Uganda Virus Research Institute, Nakiwogo, Entebbe Uganda; 3 CHF International, Nairobi, Kenya; 4 Philip R. Lee Institute for Health Policy Studies, University of California, San Francisco San Francisco, California USA; 5 Department of Sociology, Syracuse University, NY, USA; 6 Department of Microbiology and Immunology, State University of New York Upstate Medical University, Syracuse, New York USA

## Abstract

**Background:**

Malaria is a leading global cause of preventable morbidity and mortality, especially in sub-Saharan Africa, despite recent advances in treatment and prevention technologies. Scale-up and wide distribution of long-lasting insecticide-treated nets (LLINs) could rapidly decrease malarial disease in endemic areas, if used properly and continuously. Studies have shown that effective use of LLINs depends, in part, upon understanding causal factors associated with malaria. This study examined malaria beliefs, attitudes, and practices toward LLINs assessed during a large-scale integrated prevention campaign (IPC) in rural Kenya.

**Methods:**

Qualitative interviews were conducted with 34 IPC participants who received LLINs as part of a comprehensive prevention package of goods and services. One month after distribution, interviewers asked these individuals about their attitudes and beliefs regarding malaria, and about their use of LLINs.

**Results:**

Virtually all participants noted that mosquitoes were involved in causing malaria, though a substantial proportion of participants (47 percent) also mentioned an incorrect cause in addition to mosquitoes. For example, participants commonly noted that the weather (rain, cold) or consumption of bad food and water caused malaria. Regardless, most participants used the LLINs they were given and most mentioned positive benefits from their use, namely reductions in malarial illness and in the costs associated with its diagnosis and treatment.

**Conclusions:**

Attitudes toward LLINs were positive in this rural community in Western Kenya, and respondents noted benefits with LLIN use. With improved understanding and clarification of the direct (mosquitoes) and indirect (e.g., standing water) causes of malaria, it is likely that LLIN use can be sustained, offering effective household-level protection against malaria.

## Background

Malaria is one of the most significant global determinants of illness, death, and poverty [1], and has especially impacted health in the sub-Saharan African region [2]. Approximately half of the world's population is at risk of malaria, and one million deaths were attributable to malaria in 2006, with 90 percent of those deaths occurring in Africa [1]. With advances in medical therapy [3] and prevention interventions, such as bed nets, malaria is increasingly both preventable and treatable [4]. Long-lasting insecticide-treated nets (LLINs) serve as a protective barrier against mosquito bites and have been found to be a highly-effective method for preventing malaria [5].

Kenya is especially impacted by malaria accounting for a disproportionate burden of the disease[4]. Though the proportion of children and pregnant women who regularly sleep under LLINs in Kenya has increased dramatically in recent years, with a reported decrease in disease burden attributable to malaria [6], insecticide-treated nets are not fully used in Kenya, particularly in Western Kenya. According to the 2008-9 Kenya Demographic and Health Survey, 71.4 percent of households in Western Province currently own an insecticide-treated net (ITNs), and but only about half (55.4 percent) of children slept the prior night under an ITN [7]. As such, Western Province falls short of the Africa-wide goal of 80 percent of children sleeping under ITNs by 2010, and also demonstrates two gaps: 1) about one-quarter to one-third of households in Western Province lack ITNs, and 2) about one-third of children in households with ITNs do not have access to sleeping under them. Increasing supply of ITNs to households in Western Kenya could potentially reduce both gaps, and indeed supporting expansion of ITNs in Kenya is one of the main strategic approaches promoted by the Kenya Ministry of Health in its National Malaria Strategy (2001-2010) [8]. Similarly, especially in sub-Saharan Africa, countries will struggle and likely will not reach the 2015 Millennium Development Goal to halt and reverse progress in malaria incidence [9], due to difficulties with both scaling-up and proper use of these interventions [10].

Despite growing efforts at the global level to increase malaria knowledge [11], many communities throughout the world lack even a rudimentary understanding of the natural history of malaria, its relationship with mosquitoes, and the mechanisms for its treatment and prevention[12]. Understanding malaria's aetiology and relationship with the mosquito's life cycle is essential to properly implement prevention strategies [13] and effective treatment regimens [14]. Some studies have shown that even when prevention and treatment are accessible, some populations do not effectively implement those strategies, in part due to their misunderstanding of the causes of malaria [15] and to social-behavioural barriers to using products like bed nets [16]. Uptake in procurement and use of bed nets in Kenya is challenging, and modalities of distribution other than free mass distribution have not generally improved equity of access [17]. Further, Chuma *et al *showed that access and use of insecticide-treated nets in Kenya is a complex combination of supply chain difficulties, social preferences, and beliefs, going beyond simply affordability and cost [18]. Additionally, Widmar *et al *demonstrated in Tanzania that use of insecticide-treated nets requires reinforcement of proper practice regarding care and maintenance of the nets and attitudes toward their use [19]. Undoubtedly, understanding local circumstances, knowledge, and reactions to nets and other interventions is essential for building sustained action against malaria.

With the goal of rapidly improving one rural Kenyan population's ability to prevent malaria, this study examined beliefs and attitudes toward malarial illness, its prevention, and in particular the use of long-lasting insecticide-treated nets (LLINs) distributed in a mass campaign.

## Methods

The LLINs were distributed as part of an integrated prevention campaign (IPC) organized over one week's time in Lurambi Division of Kakamega District, Western Province, Kenya, in September 2008. Lurambi is a poor, inland farming area of the district, where approximately three-fourths of the population subsisted on less than $0.50 per day in 2004 [20]. Approximately 47,000 people from the region attended the campaign, which included the following components: HIV voluntary counseling and testing (VCT), distribution of a bundled package of interventions (condoms, long-lasting insecticidal-treated nets (LLINs), water filters (LifeStraw^® ^Family or Personal), and cotrimoxazole prophylaxis for those with HIV), and health education and advice. The campaign was conceived by Vestergaard Frandsen, adapting past campaign approaches. A team of local, national, and global public and private sector partners, including the Kenya Ministry of Health and the Kenya Medical Research Institute, implemented IPC. Additional details of the overall campaign and the IPC procedures can be found elsewhere [21].

The qualitative assessment of the IPC addressed participants' perceptions of malaria and its causes, LLIN practice, and perceptions of benefits and risks of using LLINs. In total, 34 in-depth qualitative interviews were conducted two months post-campaign. Individuals participating in the campaign were informed about the study as they left the product distribution point and, if they consented to participate, were registered for inclusion and their locator information taken. At the start of the interview, participants were informed of the study purpose, reassured of confidentiality of information divulged, and verbally consented to be interviewed. Local interviewers underwent thorough training on asking sensitive questions and also signed confidentiality agreements before the study commenced.

The qualitative interviews lasted an average of nearly 1.5 hours and were audio-taped, transcribed and translated from Kiswahili (82 percent of the interviews) and local languages (nine percent) into English. Text sections were coded by a pair of experienced social scientists and analysed using NVivo 8 (QSR International, Cambridge, MA). Code lists were developed both from study objectives and from the data. Specific codes related to this topic were then selected and text examined for recurring themes and to find illustrative quotes to elaborate on specific findings from the analysis [22]. Approximately half of the participants were female, with an average age of 38 years. Most participants were farmers with some secondary school education.

All participants provided informed consent. The Institutional Review Board of the Kenya Medical Research Institute (KEMRI) approved the study.

## Results

### Causes of malaria

**Figure 1:**
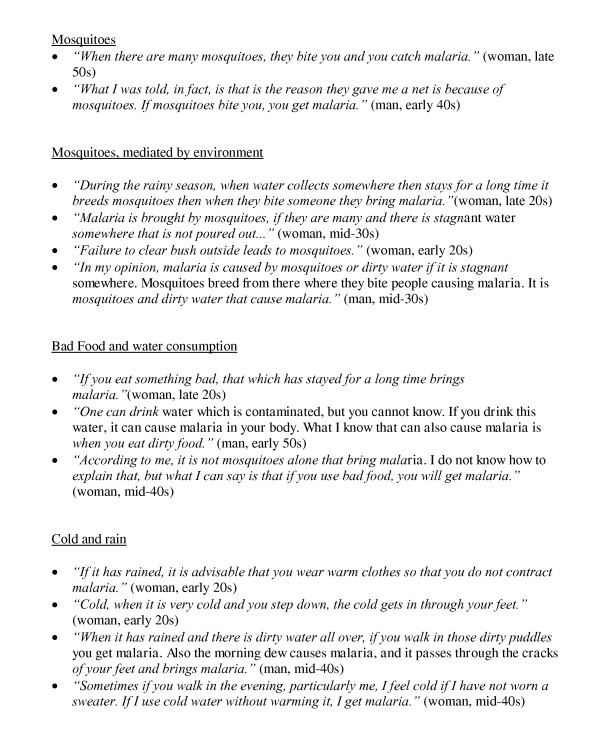
**Malaria Causation Beliefs (self), Qualitative Responses**.

As shown in Table 1 all but one participant indicated that they believed mosquitoes were at least one of the causes of malaria, and for 15 participants (44 percent), mosquitoes were, in fact, named as the only cause of malaria. For other participants, however, several mediating factors were listed along with mosquitoes as causing malaria. For example, nine participants believed that mosquitoes combined with environmental factors related to shrubbery, standing water, and pollution provided more complete explanations for the cause of malaria, sometimes with a detailed and accurate description of the role of such factors in the mosquito's life cycle (see Figure 1). As displayed in Figure 1 several participants noted that standing or stagnant water creates an environment that breeds mosquitoes. Additionally, a few participants indicated an inaccurate attribution of the environment, particularly the role of pollution and "dirt":

**Table 1:** Malaria causation beliefs among participants, and among other villagers as described by participants, IPC, Kenya

Malaria is caused by:	Total (N = 34)
**Self**^1^	
	
Mentioned mosquitoes as cause	33 (97.1%)
Mentioned environmental cause^2^	9 (26.5%)
Mentioned bad food/water consumption as cause	8 (23.5%)
Mentioned cold temperatures/rain as cause	13 (38.2%)
	
Mentioned mosquitoes solely as cause	15 (44.1%)
Mentioned mosquitoes plus additional cause	18 (52.9%)
Mentioned both an accurate and an inaccurate cause	16 (47.1%)
	
**Others**	
	
Mentioned mosquitoes as cause	20 (58.8%)
Mentioned environmental cause	5 (14.7%)
Mentioned bad food/water consumption as cause	6 (17.6%)
Mentioned cold temperatures/rain as cause	6 (17.6%)
	
Mentioned mosquitoes solely as cause	14 (41.2%)
Mentioned mosquitoes plus additional cause	6 (17.6%)
Did not mention moquitoes; mentioned other cause	9 (26.5%)
Did not know cause	5 (14.7%)

^1^Participants could mention more than one cause

^2^Environmental cause: pollution, standing water, garbage, dirty, brush

*"Malaria is caused by dirt that is thrown about outside." *(man, late 40s)

*"Malaria is brought about by dirty things." *(woman, late 40s)

Two specific components of weather (cold and rain) were commonly perceived as causes of malaria (in addition to mosquitoes) by 13 (38 percent) participants, and often characterized (Figure 1) as the type of cold requiring warm clothing, or a dampness that enters the body through exposed feet. Finally, eight participants (24 percent) indicated that consumption of bad food or water can - sometimes along with exposure to mosquitoes - cause malaria, though as shown in Figure 1 participants do not generally implicate specific foods beyond "bad" and "dirty" in this explanation.

Overall, 16 participants (47 percent), while indicating that mosquitoes caused malaria, also indicated that some other cause - such as cold temperature, rain, consumption of bad food - causes malaria, independent of those items' relationships to mosquito breeding conditions (for instance, "water" is implicated causally in local notions of malaria beyond its role as a breeding condition, for example in consumption).

When asked what other people in their villages believed about the causes of malaria, participants were less clear and were less likely to name mosquitoes as part of what the respondents perceive that other people think causes malaria. Mosquitoes, however, remained the most commonly named cause attributed to others; other categories named included environmental elements, weather, and consumption of bad food and water:

*"Eating of young maize which has a lot of water they think brings malaria, also this other newly harvested food, that is what they think." *(woman, late 50s)

*"According to the people in Lurambi, the cause of malaria is dirty environment and also dirty waste water and water that is not treated." *(man, mid-40s)

*"It is mostly mosquito bites and cold weather. It reaches a time when the weather is too cold then malaria gets its way into the body." *(man, early 40s)

*"Dirty water brings malaria. Using dirty utensils and other things too. It is grass that brings mosquito." *(woman, early 20s)

Several respondents, however, indicated that while these conceptions were common in former times, now most people knew that mosquitoes caused malaria:

*"Previously they have been thinking of different things, maybe they say, they have drunk water somewhere. They used to think that drinking water in some places can cause malaria. Sometimes they have thought that if you have new harvests and the moment they start testing the new harvest they used to think that you get malaria. But now they know that it is only mosquitoes that can cause malaria." *(man, early 50s)

*"You know, there's that talk that people like telling their children that when they play in the rain they will get malaria, the say cold rain. But when we go for the lessons, we are told that cold can't cause malaria." *(woman, early 40s)

In some cases, misguided beliefs regarding malaria causation led participants to report actions or other concepts that did not relate to the biomedical understanding of malaria:

*"You see, sometimes you could make love to your husband and after that you feel your body has malaria. You then start feeling what has come to me." *(woman, mid-40s)

*"One way of protecting myself is to avoid moving in company of people who are involved in bad activities. We have drunkards and smokers who are not using protective package that was given. Anyone who uses what was given cannot get malaria." *(man, late 40s)

Additionally, one participant who reported not currently using the LLIN responded when asked "What do you do to protect against malaria" said:

*"We used to boil water, put in a clean pot and cover it with a piece of cloth. Once the water cools we used to sieve it before pouring the water in another clean pot for drinking*. [Interviewer: What are you doing to protect yourself against malaria:] *Currently, we just boil our water for drinking because we were not given a water filter." *(woman, mid-30s)

### LLIN practice and attitudes

As shown in Table 2 overall 20 participants (59 percent) had LLINs before this campaign. After the campaign, 30 participants (88 percent) used the LLIN received from the campaign themselves, with another three participants indicating that someone else (their children or grandchildren) was using the new LLIN they received.

**Table 2:** Malaria prevention practice by participants, IPC, Kenya

Prevention Practice	Total (N = 34)
**Had a Bed net Before this Campaign**	20 (58.8%)
	
**How do you protect against malaria?**	
	
Use bed net	32 (94.1%)
Clean up household environment	3 (8.8%)
Eliminate standing water	3 (8.8%)
Eliminate mosquitoes	2 (5.9%)
Use tablets or drugs	6 (17.6%)
Something else	3 (8.8%)
	
**Who uses the net received from campaign?**	
	
Self	30 (88.2%)
Other	3 (8.8%)
Not mentioned	1 (2.9%)
	
**Benefits of using a bed net**	
	
Does not get sick from malaria anymore	23 (67.6%)
Keeps mosquitoes and bugs away	14 (41.2%)
Warmth	6 (17.6%)
Mentioned something positive	30 (88.2%)
	
**Mentioned complications of using bed net**	
	
Self	2 (5.9%)
In others	2 (5.9%)

*"I am still using the mosquito net. I also used to use some tablets for treatment of malaria but I am currently using a mosquito net, cleaning my compound, and avoiding stagnant water." *(man, mid-40s)

*"Before we had the health campaign, I in person had already had a net. The family was also using a net to prevent malaria. I was also trying to get people educated to clear around their places so that we avoid mosquitoes harboring there we were also using insecticides to spray in the houses, mosquito coils and at least clearing the bush around where we stay. And also destroying where we feel that mosquitoes can breed. In one way it was helping. Because of poverty people were not doing it the way it is supposed to be done. But it was at least helping in one way." *(man, early 50s)

**Figure 2:**
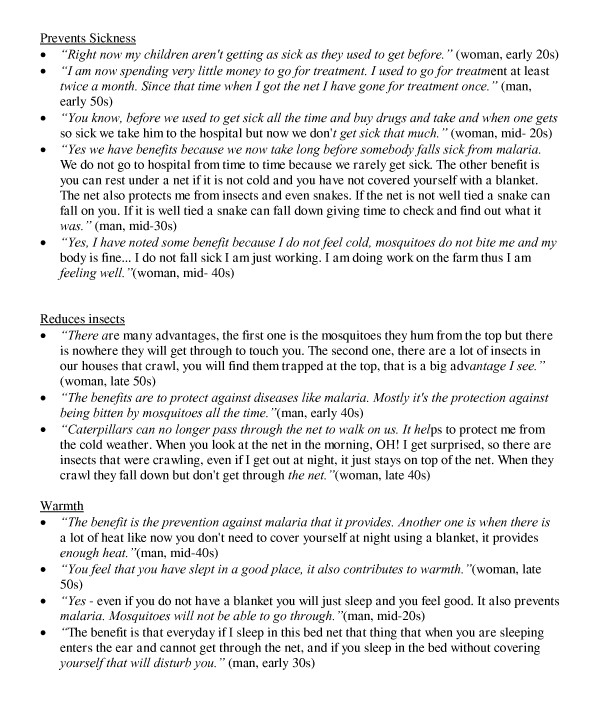
**Long-lasting Insecticide-treated nets (LLINs) benefits**.

Participants reported benefits from using the LLIN. In total, shown in Table 2, 88 percent of all participants (n = 30) reported a positive benefit, most commonly that they and their family did not get sick from malaria any more (68 percent). For instance, participants frequently mentioned that they no longer became sick with malaria when using the LLINs, and that costs associated with malaria treatment previously were reduced or eliminated (see Figure 2). In addition to noting reduced malarial illness through use of LLINs, participants also commonly mentioned that protection from insects (and other creatures) while sleeping was an additional LLIN benefit. Further, participants also commonly note that they and others feel more comfortable and "warmer" when sleeping under an LLIN, as detailed in Figure 2.

Two participants reported they experienced a minor adverse reaction themselves in using the LLIN, both relating to skin reactions:

*"The contact of the mosquito net made me itch before I followed the instructions." *(man, mid-40s)

*"The problem was at the time I was given it, it was written on the net that I finish 24 hrs. The time I opened and removed it was at night. When I did that I started scratching myself. That is when I was told that I got to see the instructions. The instructions were that I leave it for 24 hours before I cover myself." *(woman, mid-40s)

Additionally, two participants reported that they heard of others who experienced complications:

*"But some of my friends who got those mosquito nets were saying the nets had got some drugs in them that affected their stomachs. That they felt bad in their stomachs when using the net. I have personally not experienced that. In my house there has been no such a thing and even my wife has not complained." *(man, early 50s)

*"Other people just said that when they used it their bodies were itching but I did not feel itchy, no, I followed the instructions I was given." *(woman, late 40s)

### Suggestions to increase LLIN usage

Finally, as participants commonly noted that LLINs were helpful and that their use should increase, a variety of suggestions provided ideas about how best to increase LLIN usage, including:

#### Continue to increase LLIN awareness

Participants suggested several ideas around increasing LLIN awareness and their utility:

"People should be told the uses of the net when they go to the hospital to seek treatment, also they can be told when they go to church."

"I advise them if they received a bed net they should sleep under it because it reduces malaria and mosquito bites."

"There are people who get malaria but they don't know why they should use a net. They should be convinced to use them and they will provide themselves the goodness of using the net. They should be told the truth about the use of the net. Some are not aware how they should us it even it if is possible, the way you people are coming back, you need to tell them to use the net."

#### LLIN champions

Some participants suggested that having "champions" or "witnesses" promoting LLIN use would be effective, and offered specific examples for programme planners:

"By those examples, like me, I can get my friend and tell him or her that when I started using the net, I have ceased to get malaria so it is better for you to use a net, if possible."

"Acting as the example I will make them to admire according to my illustration of how I am safe by using a net. I would love you to advise and also tell those people that already have nets to be witnesses and give advice to those who don't want to use the net."

"Maybe people should be told that the best way to stop malaria is by using the net, also they should be given nets for free and be told to be taking them for treatment for free. I think this will make them use nets every time."

"They [others who don't use nets] need to be told to use it because it helps us so much, there is no malaria on the children."

Finally, participants discussed the perception that LLIN recipients were obligated to use them ("*If you were given one, then you need to use it") *and also the perceived intransigence of resistance to using LLINs if someone opposes them:

"The clever ones use it, only a few of them don't use it. Trying to convince those ones to use it is like "playing a guitar to a goat" if they don't want to use it they will never appreciate the fact that it is useful, even if you tell them."

Though not designed as a quantitative assessment, this study did not detect statistical variation in comments by gender or age.

## Discussion

Access to and proper use of LLINs are main components of effective malaria control in sub-Saharan Africa. Many countries have started scaling up free or subsidized provision of bed nets, though overall coverage remains short of the WHO goal of reaching at least 80 percent of those at risk of or suffering from malaria [1]. The distribution of bed nets coupled with social marketing has proven able to achieve proper usage by the population [6]. Such campaigns educated the population so that nets were not resold, were used properly, and were maintained effectively [23]. Success in reaching the MDG Goal 6 and other global malaria goals depend upon educating populations regarding the health value of using LLINs and on providing access to effective nets [10].

Lurambi, the setting for this present study in Western Province, is one of the most impoverished communities in Kenya, with substantial social inequity as demonstrated by the GINI index [24]. Prior experience suggests in such communities, free mass distribution of bed nets is the recommended approach to improve access and equity of access to malaria prevention interventions [17]. Accounting for local beliefs and resulting practices around use of bed nets is therefore important to help promote maximum prevention capacity in this community. Conceptual models of malaria among villagers reflected conflated understandings, particularly surrounding water, mosquitoes, and pollution, as well as notions of the impact of temperature and weather on malarial disease. Specifically, participants were aware that mosquitoes were important in the transmission of malaria, and that bed nets provided protection from mosquitoes conferring the benefit of malaria prevention. Those correct ideas, however, around mosquitoes co-existed with other notions about malaria being caused by cold temperatures, rain, and consuming bad food and water, all of which may lead to other precautionary measures that may or may not affect actual malarial risk.

As has been shown elsewhere in Africa, ideas around causation and transmission of malaria could be associated with discontinuation or suboptimal use of LLINs if populations do not believe that LLINs are important prevention mechanisms based on their belief systems and world views [15]. Given the deep historical context within which populations co-evolved along with malaria and the mosquitoes that maintain its transmission, it is understandable that notions around weather, pollution, temperature, and spoiled food and water consumption have developed as causes of malaria, since malaria accompanies rainy seasons and prevention messages often stress the importance of eliminating water as a factor in the environment. As demonstrated in this present study, however, some villagers believe that strategies such as boiling water can prevent malaria, which although a healthy public health practice in areas where water quality is questionable, will not impact malaria incidence. It is unclear whether or not such statements of belief represent misunderstanding of multiple campaign messages addressing different preventive topics (of which clean water and prevention of water-borne illness was one).

The study was limited in several ways. First, while redundancy was reached with participants on salient issues, sample size was small and may not reflect the range of variation in response on all topics. Secondly, as a qualitative study, findings are limited to identifying concepts and models rather than quantifying magnitude of variables. As such, this study may not necessarily be directly generalizable across populations.

The findings of this study could potentially be used to improve proper bed net usage and other prevention strategies around malaria. First, the study showed that aetiologically correct biomedical perceptions of malaria co-exist with more traditional beliefs around the impact of cold temperature and efforts can be made to continue to promote proper understanding of malaria, the role of mosquitoes, and the importance of creating a physical barrier against them. Secondly, participants demonstrated that bed nets are acceptable and generally used, endorsing Norr *et al*'s findings [17] that once cost as a barrier to access is removed, impoverished populations in Kenya can successful adopt bed nets as a prevention strategy. Finally, additional other benefits of bed net usage mentioned from the population's perspective should be promoted as motivations for consistent and widespread use - savings in medical and other expenses associated with malaria care, extra warmth, and barrier to other insects (and snakes) while sleeping - all could reinforce the use of bed nets within the household.

## Conclusions

Regardless of villagers' perceptions of malarial disease, LLINs were viewed positively by the participants and were directly credited by their users with reduced malarial disease and other benefits, such as warmth and protection from insects disturbing their sleep. Most participants used the LLINs they received themselves, with few complications or difficulties. Providing scaled-up access to LLINS for populations at-risk of malaria clearly could rapidly offer protection from preventable disease and mortality; further, their usage could likely be sustained with clear education about malaria's causes and contributing factors.

## Competing interests

Vestergaard-Frandsen manufactures the long-lasting insecticide-treated bed nets that were donated as part of the Integrated Prevention Campaign. Beyond funding, Vestergaard-Frandsen did not participate in analysis or manuscript development except to ensure accurate description of the Campaign and the products distributed within it. The authors declare that they have no competing interests.

## Authors' contributions

TD was principal analyst and primary author of this manuscript. RA and EL designed and carried out the qualitative data collection. JGK, JS, and CO drafted sections of the manuscript. All authors read and approved the final manuscript.
